# 2-Chloro-3-hydroxy­methyl-7,8-dimethyl­quinoline

**DOI:** 10.1107/S160053680905404X

**Published:** 2009-12-19

**Authors:** F. Nawaz Khan, S Mohana Roopan, Venkatesha R. Hathwar, Seik Weng Ng

**Affiliations:** a Chemistry Division, School of Science and Humanities, VIT University, Vellore 632 014, Tamil Nadu, India; b Solid State and Structural Chemistry Unit, Indian Institute of Science, Bangalore 560 012, Karnataka, India; c Department of Chemistry, University of Malaya, 50603 Kuala Lumpur, Malaysia

## Abstract

All non-H atoms of the title compound, C_12_H_12_ClNO, are co-planar (r.m.s. deviation = 0.055 Å). The hydr­oxy H atom is disordered over two positions of equal occupancy. In the crystal, mol­ecules are linked by O—H⋯O hydrogen bonds, generating zigzag chains running along the *b* axis.

## Related literature

Substituted quinoline-3-carbaldehydes are inter­mediates for annelation and functional group modification; for a review of the synthesis of quinolines by the Vilsmeier–Haack reaction, see: Meth-Cohn (1993 ▶).
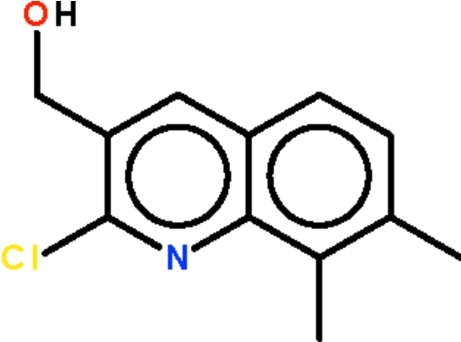


## Experimental

### 

#### Crystal data

C_12_H_12_ClNO
*M* *_r_* = 221.68
Monoclinic, 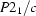

*a* = 17.4492 (12) Å
*b* = 4.6271 (2) Å
*c* = 14.3773 (7) Å
β = 113.297 (7)°
*V* = 1066.17 (10) Å^3^
*Z* = 4
Mo *K*α radiation
μ = 0.33 mm^−1^
*T* = 293 K
0.38 × 0.15 × 0.06 mm

#### Data collection

Bruker SMART area-detector diffractometer
Absorption correction: multi-scan (*SADABS*; Sheldrick, 1996 ▶) *T*_min_ = 0.885, *T*_max_ = 0.981
10456 measured reflections
1884 independent reflections
1488 reflections with *I* > 2σ(*I*)
*R*_int_ = 0.033

#### Refinement

*R*[*F* ^2^ > 2σ(*F* ^2^)] = 0.033
*wR*(*F* ^2^) = 0.094
*S* = 1.05
1884 reflections
139 parameters
H-atom parameters constrained
Δρ_max_ = 0.16 e Å^−3^
Δρ_min_ = −0.22 e Å^−3^

### 

Data collection: *SMART* (Bruker, 2004 ▶); cell refinement: *SAINT* (Bruker, 2004 ▶); data reduction: *SAINT*; program(s) used to solve structure: *SHELXS97* (Sheldrick, 2008 ▶); program(s) used to refine structure: *SHELXL97* (Sheldrick, 2008 ▶); molecular graphics: *X-SEED* (Barbour, 2001 ▶); software used to prepare material for publication: *publCIF* (Westrip, 2010 ▶).

## Supplementary Material

Crystal structure: contains datablocks global, I. DOI: 10.1107/S160053680905404X/bt5139sup1.cif

Structure factors: contains datablocks I. DOI: 10.1107/S160053680905404X/bt5139Isup2.hkl

Additional supplementary materials:  crystallographic information; 3D view; checkCIF report

## Figures and Tables

**Table 1:** Hydrogen-bond geometry (Å, °)

*D*—H⋯*A*	*D*—H	H⋯*A*	*D*⋯*A*	*D*—H⋯*A*
O1—H1a⋯O1^i^	0.82	1.91	2.715 (3)	167
O1—H1b⋯O1^ii^	0.82	1.91	2.720 (3)	168

Symmetry codes: (i) 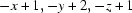; (ii) 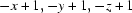.

**Fig. 1.**
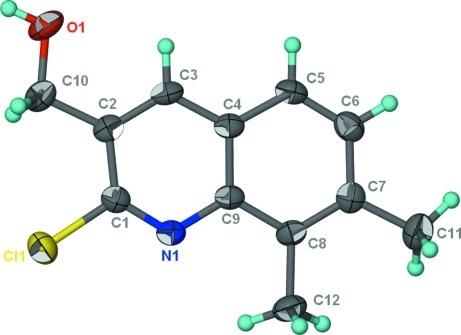
Thermal ellipsoid plot (Barbour, 2001) of C12H12ClNO at the 50% probability level; hydrogen atoms are drawn as spheres of arbitrary radius.

## supplementary crystallographic information

### Experimental

2-Chloro-7,8-dimethylquinoline-3-carbaldehyde (220 mg, 1 mmol), sodium borohydride (38 mg, 1 mmol) and catalytic amount of montmorillonite K-10 were placed in a beaker. The contents were irradiated at 500 W for 5 min. The product was dissolved in ethyl acetate and the residue removed by filtration. The filtrate was subjected to column chromatography on silica, and ethyl acetate/petroleum ether was used as the eluant. The solvent was evaporated and the residue recrystallized from chloroform to give colorless crystals.

### Refinement

Hydrogen atoms were placed in calculated positions (C–H 0.93–0.97, O–H 0.82 Å) and were included in the refinement in the riding model approximation, with *U*(H) set to 1.2–1.5*U*(C,*O*). The methyl H-atoms were refined as disordered over two equally occupied sites. The hydroxy H-atom is also disordered over two positions with equal site occupancy.

### Crystal data

**Table:**

C_12_H_12_ClNO	*F*(000) = 464
*M**_r_* = 221.68	*D*_x_ = 1.381 Mg m^−^^3^
Monoclinic, *P*2_1_/*c*	Mo *K*α radiation, λ = 0.71073 Å
Hall symbol: -P 2ybc	Cell parameters from 963 reflections
*a* = 17.4492 (12) Å	θ = 3.1–25.0°
*b* = 4.6271 (2) Å	µ = 0.33 mm^−^^1^
*c* = 14.3773 (7) Å	*T* = 293 K
β = 113.297 (7)°	Plate, colorless
*V* = 1066.17 (10) Å^3^	0.38 × 0.15 × 0.06 mm
*Z* = 4	

### Data collection

**Table:**

Bruker SMART area-detector diffractometer	1884 independent reflections
Radiation source: fine-focus sealed tube	1488 reflections with *I* > 2σ(*I*)
graphite	*R*_int_ = 0.033
φ and ω scans	θ_max_ = 25.0°, θ_min_ = 3.1°
Absorption correction: multi-scan (*SADABS*; Sheldrick, 1996)	*h* = −20→20
*T*_min_ = 0.885, *T*_max_ = 0.981	*k* = −5→5
10456 measured reflections	*l* = −17→17

### Refinement

**Table:**

Refinement on *F*^2^	Primary atom site location: structure-invariant direct methods
Least-squares matrix: full	Secondary atom site location: difference Fourier map
*R*[*F*^2^ > 2σ(*F*^2^)] = 0.033	Hydrogen site location: inferred from neighbouring sites
*wR*(*F*^2^) = 0.094	H-atom parameters constrained
*S* = 1.05	*w* = 1/[σ^2^(*F*_o_^2^) + (0.0496*P*)^2^ + 0.1434*P*] where *P* = (*F*_o_^2^ + 2*F*_c_^2^)/3
1884 reflections	(Δ/σ)_max_ = 0.001
139 parameters	Δρ_max_ = 0.16 e Å^−^^3^
0 restraints	Δρ_min_ = −0.22 e Å^−^^3^

### Special details

**Table:**

Geometry. All e.s.d.'s (except the e.s.d. in the dihedral angle between two l.s. planes) are estimated using the full covariance matrix. The cell e.s.d.'s are taken into account individually in the estimation of e.s.d.'s in distances, angles and torsion angles; correlations between e.s.d.'s in cell parameters are only used when they are defined by crystal symmetry. An approximate (isotropic) treatment of cell e.s.d.'s is used for estimating e.s.d.'s involving l.s. planes.

### Fractional atomic coordinates and isotropic or equivalent isotropic displacement parameters (Å^2^)

**Table:**

	*x*	*y*	*z*	*U*_iso_*/*U*_eq_	Occ. (<1)
Cl1	0.37792 (3)	0.62190 (11)	0.12385 (3)	0.0547 (2)	
O1	0.46228 (9)	0.7503 (3)	0.45560 (9)	0.0578 (4)	
H1A	0.4892	0.8991	0.4763	0.087*	0.50
H1B	0.4914	0.6102	0.4824	0.087*	0.50
N1	0.27105 (9)	0.2833 (3)	0.15172 (10)	0.0365 (3)	
C1	0.33144 (11)	0.4587 (4)	0.19891 (12)	0.0361 (4)	
C2	0.36337 (11)	0.5327 (3)	0.30325 (12)	0.0365 (4)	
C3	0.32309 (11)	0.4071 (3)	0.35710 (12)	0.0381 (4)	
H3	0.3403	0.4493	0.4256	0.046*	
C4	0.25620 (10)	0.2154 (4)	0.31127 (12)	0.0349 (4)	
C5	0.21258 (12)	0.0807 (4)	0.36385 (13)	0.0427 (5)	
H5	0.2273	0.1186	0.4323	0.051*	
C6	0.14924 (12)	−0.1037 (4)	0.31451 (13)	0.0440 (5)	
H6	0.1210	−0.1899	0.3502	0.053*	
C7	0.12436 (11)	−0.1703 (4)	0.21072 (13)	0.0398 (4)	
C8	0.16551 (11)	−0.0432 (4)	0.15657 (12)	0.0370 (4)	
C9	0.23169 (10)	0.1536 (3)	0.20687 (12)	0.0333 (4)	
C10	0.43655 (11)	0.7321 (4)	0.34903 (13)	0.0454 (5)	
H10A	0.4825	0.6623	0.3334	0.054*	
H10B	0.4215	0.9231	0.3196	0.054*	
C11	0.05264 (12)	−0.3765 (4)	0.16298 (16)	0.0558 (5)	
H11A	0.0510	−0.4393	0.0986	0.084*	0.50
H11B	0.0013	−0.2809	0.1535	0.084*	0.50
H11C	0.0599	−0.5409	0.2064	0.084*	0.50
H11D	0.0238	−0.4015	0.2071	0.084*	0.50
H11E	0.0735	−0.5598	0.1521	0.084*	0.50
H11F	0.0149	−0.2998	0.0993	0.084*	0.50
C12	0.14234 (13)	−0.1070 (4)	0.04636 (13)	0.0516 (5)	
H12A	0.1910	−0.1694	0.0363	0.077*	0.50
H12B	0.1203	0.0644	0.0073	0.077*	0.50
H12C	0.1009	−0.2568	0.0250	0.077*	0.50
H12D	0.0838	−0.0718	0.0095	0.077*	0.50
H12E	0.1545	−0.3056	0.0384	0.077*	0.50
H12F	0.1739	0.0156	0.0207	0.077*	0.50

### Atomic displacement parameters (Å^2^)

**Table:**

	*U*^11^	*U*^22^	*U*^33^	*U*^12^	*U*^13^	*U*^23^
Cl1	0.0522 (3)	0.0662 (4)	0.0492 (3)	−0.0064 (2)	0.0237 (2)	0.0071 (2)
O1	0.0632 (9)	0.0508 (8)	0.0438 (7)	−0.0109 (7)	0.0045 (6)	−0.0114 (6)
N1	0.0374 (9)	0.0395 (8)	0.0307 (7)	0.0031 (7)	0.0116 (6)	0.0004 (6)
C1	0.0360 (10)	0.0362 (10)	0.0357 (9)	0.0056 (8)	0.0138 (8)	0.0032 (7)
C2	0.0369 (10)	0.0313 (9)	0.0366 (9)	0.0060 (8)	0.0094 (8)	−0.0016 (7)
C3	0.0420 (11)	0.0384 (10)	0.0283 (8)	0.0051 (8)	0.0078 (7)	−0.0053 (7)
C4	0.0375 (10)	0.0344 (9)	0.0308 (8)	0.0068 (8)	0.0113 (7)	−0.0004 (7)
C5	0.0478 (11)	0.0506 (12)	0.0310 (9)	0.0050 (9)	0.0169 (8)	0.0009 (8)
C6	0.0431 (11)	0.0482 (11)	0.0438 (10)	0.0061 (9)	0.0203 (8)	0.0099 (8)
C7	0.0345 (10)	0.0368 (10)	0.0439 (10)	0.0062 (8)	0.0110 (8)	0.0054 (8)
C8	0.0370 (10)	0.0361 (10)	0.0320 (9)	0.0055 (8)	0.0075 (7)	0.0004 (7)
C9	0.0351 (10)	0.0335 (9)	0.0293 (8)	0.0060 (8)	0.0105 (7)	0.0007 (7)
C10	0.0448 (12)	0.0382 (10)	0.0463 (10)	−0.0017 (9)	0.0107 (8)	−0.0033 (8)
C11	0.0454 (12)	0.0536 (13)	0.0631 (13)	−0.0041 (10)	0.0157 (10)	0.0039 (10)
C12	0.0547 (13)	0.0576 (13)	0.0342 (9)	−0.0056 (10)	0.0087 (9)	−0.0064 (8)

### Geometric parameters (Å, °)

**Table:**

Cl1—C1	1.7563 (17)	C7—C11	1.506 (3)
O1—C10	1.418 (2)	C8—C9	1.423 (2)
O1—H1A	0.8200	C8—C12	1.501 (2)
O1—H1B	0.8200	C10—H10A	0.9700
N1—C1	1.290 (2)	C10—H10B	0.9700
N1—C9	1.375 (2)	C11—H11A	0.9600
C1—C2	1.420 (2)	C11—H11B	0.9600
C2—C3	1.364 (2)	C11—H11C	0.9600
C2—C10	1.500 (2)	C11—H11D	0.9600
C3—C4	1.405 (2)	C11—H11E	0.9600
C3—H3	0.9300	C11—H11F	0.9600
C4—C5	1.412 (2)	C12—H12A	0.9600
C4—C9	1.418 (2)	C12—H12B	0.9600
C5—C6	1.354 (3)	C12—H12C	0.9600
C5—H5	0.9300	C12—H12D	0.9600
C6—C7	1.413 (2)	C12—H12E	0.9600
C6—H6	0.9300	C12—H12F	0.9600
C7—C8	1.382 (2)		
			
C10—O1—H1A	109.5	O1—C10—C2	111.18 (14)
C10—O1—H1B	109.5	O1—C10—H10A	109.4
C1—N1—C9	117.49 (14)	C2—C10—H10A	109.4
N1—C1—C2	127.22 (15)	O1—C10—H10B	109.4
N1—C1—Cl1	115.28 (12)	C2—C10—H10B	109.4
C2—C1—Cl1	117.51 (13)	H10A—C10—H10B	108.0
C3—C2—C1	114.95 (16)	C7—C11—H11A	109.5
C3—C2—C10	123.59 (15)	C7—C11—H11B	109.5
C1—C2—C10	121.46 (15)	H11A—C11—H11B	109.5
C2—C3—C4	121.44 (15)	C7—C11—H11C	109.5
C2—C3—H3	119.3	H11A—C11—H11C	109.5
C4—C3—H3	119.3	H11B—C11—H11C	109.5
C3—C4—C5	123.44 (15)	C7—C11—H11D	109.5
C3—C4—C9	118.06 (15)	C7—C11—H11E	109.5
C5—C4—C9	118.49 (16)	H11D—C11—H11E	109.5
C6—C5—C4	119.92 (16)	C7—C11—H11F	109.5
C6—C5—H5	120.0	H11D—C11—H11F	109.5
C4—C5—H5	120.0	H11E—C11—H11F	109.5
C5—C6—C7	122.47 (16)	C8—C12—H12A	109.5
C5—C6—H6	118.8	C8—C12—H12B	109.5
C7—C6—H6	118.8	H12A—C12—H12B	109.5
C8—C7—C6	119.41 (16)	C8—C12—H12C	109.5
C8—C7—C11	122.41 (16)	H12A—C12—H12C	109.5
C6—C7—C11	118.17 (16)	H12B—C12—H12C	109.5
C7—C8—C9	118.95 (15)	C8—C12—H12D	109.5
C7—C8—C12	121.87 (16)	C8—C12—H12E	109.5
C9—C8—C12	119.19 (15)	H12D—C12—H12E	109.5
N1—C9—C4	120.81 (15)	C8—C12—H12F	109.5
N1—C9—C8	118.43 (14)	H12D—C12—H12F	109.5
C4—C9—C8	120.75 (15)	H12E—C12—H12F	109.5

### Hydrogen-bond geometry (Å, °)

**Table:**

*D*—H···*A*	*D*—H	H···*A*	*D*···*A*	*D*—H···*A*
O1—H1a···O1^i^	0.82	1.91	2.715 (3)	167
O1—H1b···O1^ii^	0.82	1.91	2.720 (3)	168

Symmetry codes: (i) −*x*+1, −*y*+2, −*z*+1; (ii) −*x*+1, −*y*+1, −*z*+1.
